# Curettement of the Uterus1Read before the thirty-third annual session of the Medical Society of Virginia, held at Newport News, Va., September 23–25, 1902.

**Published:** 1903-01

**Authors:** George Ben Johnston

**Affiliations:** Richmond, Va.


### Curettement of the Uterus.[1]

By GEORGE BEN JOHNSTON, M. D., Richmond, Va.

(*Richmond Journal of Practice, September, 1902.*)

I HAVE but one purpose in presenting this brief paper and that is to utter a warning against the misuse of the uterine curette. I shall make two contentions: (1) that the practice is far too frequently resorted to; (2) that it is in many instances both useless and harmful and in some dangerous.

To support my first proposition, I quote the records of the gynecological department of one of the leading hospitals in a neighboring state, taken from the hospital report for the year 1901. There were admitted into the gynecological wards, 293 women. Of these 165 were subjected to the ordeal of curettement. It is inconceivable that 50 per cent. and over of all women presenting for gynecological treatment require curettement, and if I could furnish no other argument in support of my first proposition, this record would supply it. But I could show from my own records many cases in which the operation was unsuitable and unjustifiable and produced serious troubles, for which the patients sought my aid.

In properly chosen cases and when skilfully performed, curettement is a most valuable procedure and no other manipulation can take its place when rightly indicated, but the indications for it should be as pointed, as definite and as well understood as for any other surgical undertaking. Its application otherwise for the relief of vague and ill-defined symptoms without a clear diagnosis indicating its performance is unjustifiable and wrong.

The conditions to which it is applicable are very limited and may be enumerated as follows: (1) for purposes of diagnosis; (2) to cleanse the uterus after incomplete abortion; (3) to remove cancerous tissue in otherwise inoperable cases; (4) for the ablation of polypi and small submucous fibroids; (5) in endometritis.

The operation bears a false reputation. It is regarded as an insignificant and safe operation. This is unfortunate, for such an estimate leads to its abuse.

---

1. Read before the thirty-third annual session of the Medical Society of Virginia, held at Newport News, Va., September 23-25, 1902.

It is not an easy matter to thoroughly curette the uterus; on the contrary, great skill is required to achieve proper results. Any one may remove some diseased tissue from the cavity, but without thoroughness and completeness in the operation it becomes of no avail and for this a trained hand is essential.

Except in so far as it does not usually immediately endanger life, it is not a safe procedure, but its performance is attended by many dangers, mechanical and inflammatory. Of mechanical dangers, laceration of the cervix in the course of necessary dilatation is not uncommon and perforation of the uterus by the dilator or the curette or by the probe in packing is a serious menace. When one considers the natural uncleanliness of the field of operation and the notoriously difficult task of rendering it even approximately aseptic, it is at once evident that infection and subsequent inflammations are a threatening danger in this operation. Metritis and cellulitis are the milder inflammations met with, but it is not infrequently that we encounter the more serious forms of salpingitis and peritonitis. Undertaken in the presence of pelvic inflammation, it may light up an old inflammation or cause the further extension of one already in progress. I have seen every one of these misfortunes follow the untimely and unskilful application of the curette and have repeatedly had occasion to remove pus tubes, which were directly traceable to faulty or unnecessary curettage.

If I were called upon to formulate a set of rules governing the operation of curettement, I would announce them as follows: (1) have a distinct and sufficient reason for the procedure; (2) observe a most scrupulously perfect technique as to asepticism; (3) dilate the cervix slowly and thoroughly, under complete anesthesia; (4) apply the curette with great gentleness, but completely and thoroughly removing all diseased tissue; (5) conduct the subsequent dressings and after-treatment with the same rigid care that appertains to other surgical operations.

When applied for the purposes indicated, the results are satisfactory—even brilliant. Resorted to, however, as is often unfortunately the case, without a specific object in view, upon a vague and inadequate diagnosis and unskilfully practised, its results are disappointing and even disastrous. The operation fails or is mischievous when the reason for it is not well defined in the absence of a perfect technique and when the curettement is not thorough.

